# The Use of Single-Item Ratings Versus Traditional Multiple-Item Questionnaires to Assess Mood and Health

**DOI:** 10.3390/ejihpe11010015

**Published:** 2021-02-20

**Authors:** Joris C. Verster, Elena Sandalova, Johan Garssen, Gillian Bruce

**Affiliations:** 1 Division of Pharmacology, Utrecht Institute for Pharmaceutical Sciences, Utrecht University, 3584CG Utrecht, The Netherlands; elena.sandalova@danone.com (E.S.); j.garssen@uu.nl (J.G.); 2 Centre for Human Psychopharmacology, Swinburne University, Melbourne, VIC 3122, Australia; 3 Danone Nutricia Research, 30 Biopolis Street #05-1B/#05-2A, Matrix Building, Singapore 138671, Singapore; 4 Global Centre of Excellence Immunology, Nutricia Danone Research, 3584CT Utrecht, The Netherlands; 5 Division of Psychology and Social Work, School of Education and Social Sciences, University of the West of Scotland, Paisley PA1 2BE, UK; gillian.bruce@uws.ac.uk

**Keywords:** questionnaires, singe item ratings, assessment, real-world evidence, mobile assessments

## Abstract

Collecting real-world evidence via ‘at home’ assessments in ambulatory patients or healthy volunteers is becoming increasingly important, both for research purposes and in clinical practice. However, given the mobile technology that is frequently used for these assessments, concise assessments are preferred. The current study compared single-item ratings with multiple-item subscale scores of the same construct, by calculating the corresponding Bland and Altman 95% limits of agreement interval. The analysis showed that single-item ratings are usually in good agreement with assessments of their corresponding subscale. In the case of more complex multimodal constructs, single-item assessments were much less often in agreement with multiple-item questionnaire outcomes. The use of single-item assessments is advocated as they more often incorporate assessments of all aspects of a certain construct (including the presence, severity, and impact of the construct under investigation) compared to composite symptom scores.

## 1. Introduction

During the COVID-19 pandemic, collecting real-world evidence via ‘at home’ assessments in ambulatory patients or healthy volunteers became increasingly important, both in research and in clinical practice. Often, mobile technology is applied to collect data in real-time, without requiring hospital visits which may be more demanding in pandemic times and a burden to patients and healthcare providers [1,2]. There are, however, limitations to the design of mobile assessments [2], and the restrictions of small screens advocate for concise assessments. 

For many constructs such as sleep quality and hostility, there are no readily and easily applicable biomarkers available. Researchers, therefore, have to rely on patient-reported outcome measures (PRO) via questionnaires. It can be questioned if traditional, often lengthy, questionnaires to assess mood, quality of life, and health correlates are either necessary or practical. The use of these multiple-item questionnaires may be a burden to patients, and increase the risk of noncompletion of clinical and research assessments. Single-item assessments could be an alternative to prevent this. 

**Figure 1:**
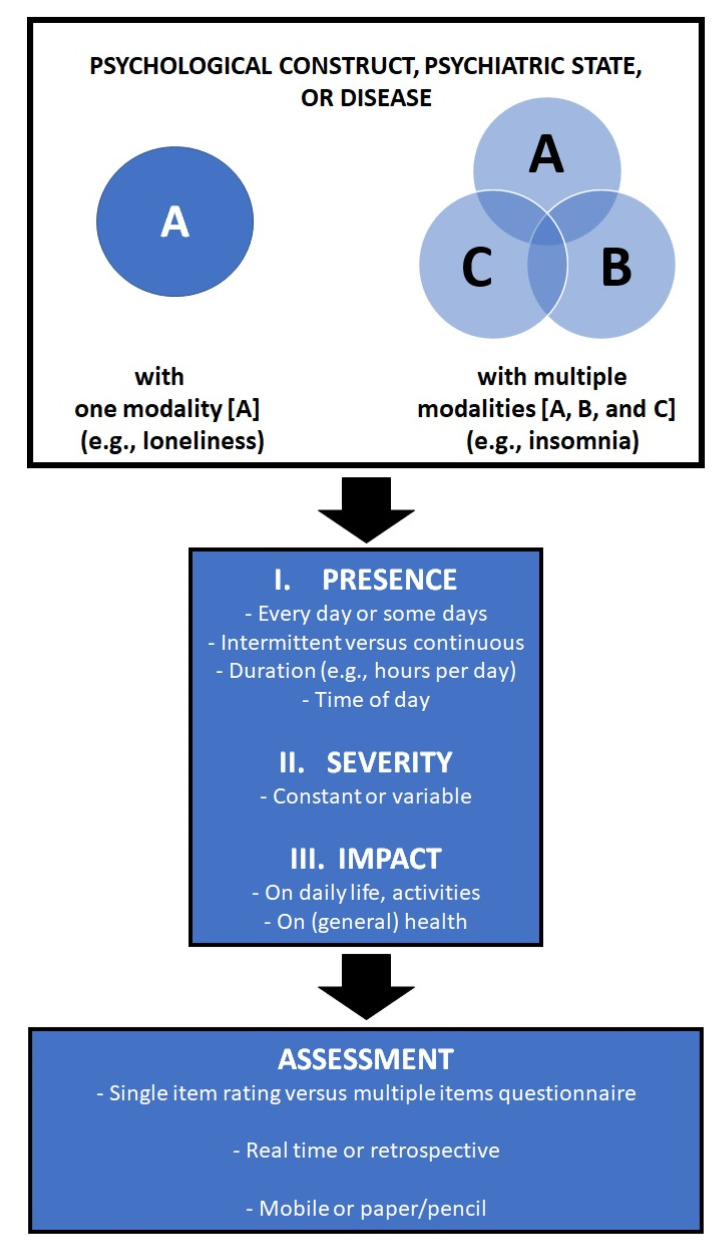
Development of a patient-reported outcome measure.

The development of a valid and reliable PRO is essential and should capture all important aspects of a condition under investigation (see Figure 1). As illustrated in Figure 1, a good PRO should evaluate the presence, severity, and impact of a condition. As such, the development of a valid PRO may not always be a straightforward venture [3]. 

Psychological constructs, psychological states, and diseases are often characterized by more than one aspect (in Figure 1 referred to as A, B, and C). For example, attention deficit hyperactivity disorder (ADHD) is characterized by inattention, hyperactivity, impulsivity; and insomnia is characterized by sleep initiation problems and/or sleep maintenance problems that have a negative impact on daytime functioning. More complex constructs such as schizophrenia, alcohol hangover, or irritable bowel syndrome may require the assessment of more individual aspects to describe the full syndrome. When a questionnaire comprises the assessment of the presence and severity of the characteristic symptoms of a disease, this either results in a lengthy questionnaire to capture all of them or in a short questionnaire that omits several symptoms that may have been of importance to individual patients. Moreover, in such questionnaires the impact on daily activities is usually not assessed.

Given this, the US Food and Drug Administration suggested that single-item assessments may even be preferred, as they incorporate the subjects’ evaluation of the presence, severity, and impact of a condition, with greater subject-focused information value than the specific symptom-based sum score of multiple-item questionnaires can provide [4]. The question, however, arises as to whether single-item assessments are equally effective as their corresponding multiple-item questionnaires. If they are equally effective, then single-item assessments would be a cost-effective and time-reducing strategy to examine mood, quality of life, and health correlates in clinical monitoring and experimental research following patients over time. The single-item assessment provides a real-time, directly available outcome. Single-item assessments could, for example, be implemented in randomized clinical trials where assessment windows relative to treatment intake are often limited, with patients for which the completion of lengthy questionnaires may be a burden (e.g., elderly), or in clinical practice to determine if a more thorough evaluation of a patient is warranted. The purpose of the current analysis was to evaluate whether assessments of single-item scales are in agreement with their corresponding multiple-item scales.

## 2. Materials and Methods

To compare single-items and multiple item scales, data from Balikji et al. [5] were re-evaluated. In this study, N = 2489 participants (83.4% women) completed an online survey on ‘food and health’. The Dutch participants, aged 18 to 30 years old, were recruited via Facebook. Their mean (standard deviation, SD) age was 21.3 (2.1) years old. For the current analysis, we re-evaluated assessments of mood, mental resilience, insomnia, and irritable bowel syndrome (IBS), which are described below. 

### 2.1. Profiles of Mood States—Short Form (POMS-SF)

The short version of the Profiles of Mood States (POMS-SF) was completed to assess mood [6,7,8]. The Dutch version comprises 32 items that are scored on a 5-point Likert scale (0 = not at all, 4 = extremely) and has five subscales assessing tension (6 items), depression (8 items), anger (7 items), fatigue (6 items), and vigor (5 items). Higher scores on the scales imply more psychological distress. In addition to the subscales, a total psychological distress score was computed as the sum score of all 32 items. For the comparison between scale scores and single-items, the items “angry” (anger), “blue” (depression), “tense” (tension), “vigorous” (vigor), “fatigued” (fatigue) were selected.

### 2.2. The Depression Anxiety Stress Scales (DASS-21)

The Dutch version of the Depression Anxiety Stress Scales (DASS-21) was completed to assess stress, anxiety, and depression [9,10,11]. The scale consists of 21 items that can be scored on a 4-point Likert scale, (0 = not at all, 3 = very much or most of the time). The sum scores are computed for three scales assessing depression (7 items), anxiety (7 items), and stress (7 items). A higher scale score is associated with a greater level of depression, anxiety, and/or stress. In addition to the subscales, a total psychological distress score was computed as the sum score of all 21 items. For the comparison between scale scores and single-items, the items “I felt down-hearted and blue” (depression), “I felt scared without any good reason” (anxiety), and “I found it difficult to relax” (stress) were selected.

### 2.3. Brief Resilience Scale (BRS)

Mental resilience was evaluated utilizing the Brief Resilience Scale [12]. The BRS comprises 6 items that assess one’s ability to recover from stress, i.e. to bounce back. The 6 items can be rated on a 5-point Likert scale ranging from ‘strongly disagree’ to ‘strongly agree’. Scores range from 1 to 5, and reversed scoring is applied to some of the items. The mean score of the six items was computed to represent the level of mental resilience. A higher score implies a higher level of mental resilience. Previous research revealed that mental resilience scores correlated significantly with personality characteristics, psychological coping strategies, and health correlates [12,13]. For the comparison between scale scores and single-items, the item “I tend to take a long time to get over set-backs in my life” was selected.

### 2.4. SLEEP-50 Insomnia Subscale 

The 9-item insomnia subscale of the SLEEP-50 questionnaire [14] was completed. Each item can be scored on a 4-point scale ranging from 1 (not at all), 2 (somewhat), 3 (rather much), and 4 (very much), and the total insomnia score is computed by adding up scores on the individual items. A total insomnia score was computed as the sum score of all 9 items. For the comparison between scale scores and single-items, the items “I have difficulty in falling asleep” (sleep initiation problems) and “After waking up during the night, I fall asleep slowly” (sleep maintenance problems) were selected. 

### 2.5. Birmingham IBS Symptom Questionnaire

The presence and severity of IBS symptoms were assessed with the Dutch version of the Birmingham IBS Symptom Questionnaire [5,15,16]. The questionnaire consists of 11 items, with 6-answer possibilities, ranging from 0 (‘none of the time) to 5 (‘all of the time’). Scores on three symptom specific scales representing the factors ‘diarrhea’ (5 items), ‘constipation’ (3 items), and ‘pain’ (3 items) were calculated. Highly scores imply more complaints. In addition to the subscales, a total IBS score was computed as the sum score of all 11 items. For the comparison between scale scores and single-items, the items “How often have you had discomfort or pain in your abdomen?” (pain), “How often have you been troubled with diarrhea?” (diarrhea), and “How often have you been troubled by constipation?” (constipation) were selected.

### 2.6. Single-Item Selection

The single-item was part of the respective subscale and was selected by the authors (J.C.V. and G.B.) based on the representativeness of an overall construct and if scores on the item had the highest correlation with the respective construct. For most subscales, this was a straightforward selection as the subscales comprised an item that was the same as the overall scale construct (e.g., the item “fatigued” of the POMS-SF fatigue scale). For other scales, based on the investigators’ judgment, an item was chosen that best represented the overall assessed construct (e.g., “I felt it difficult to relax” for the DASS21 stress subscale). The insomnia scale has no subscales. For this scale two items instead of one item were selected, to reflect that insomnia can be characterized by sleep initiation problems, sleep maintenance problems, or both.

### 2.7. Statistical Analyses

Statistical analyses were conducted with SPSS (IBM Corp. Released 2013. IBM SPSS Statistics for Windows, Version 25.0. Armonk, NY, USA: IBM Corp.). First, Spearman’s rank correlation coefficients (Rho) were computed between single-item and corresponding full-scale scores. Correlations were considered statistically significant if *p* < 0.05 (in a two-tailed test). Second, to demonstrate whether the outcomes of the single-item assessment were not different from their corresponding subscales, the 95% limits of agreement method by Bland and Altman [17] was applied. In order to make the two assessments directly comparable, the single-item scores were multiplied by the number of items of the corresponding subscale. Difference scores (DIFF) of the two outcomes (full-scale score–single-item score) and the corresponding standard deviation (SD_DIFF_) were computed. The 95% limits of agreement method states that agreement between methods (in this case single-item versus full-scale assessment) can be concluded if 95% of the DIFF scores lie between (DIFF − 1.96 × SD_DIFF_) and (DIFF + 1.96 × SD_DIFF_). In other words, the outcomes of the two assessments are significantly different if more than 5% of the differences scores lie outside the 95% limits of agreement interval. Bland and Altman plots were produced which show the difference between the single-item assessments (multiplied by the number of subscale items) and subscale assessments for each subject against the mean score of the two assessments [17]. If the assessments are identical, datapoints are close to the line of equality (zero) and 95% of the data points is lies between the lower and upper limit of the 95% limits of agreement interval. The plots also illustrate the presence of possible extreme or outlying observations. Third, applying the same Bland and Atman methodology [17] it was evaluated whether the outcomes of the single-item assessment are in agreement with the corresponding full-scale outcomes of the POMS-SF, DASS21, Birmingham IBS scale, and SLEEP-50 insomnia scale. For this comparison, the single-item scores were multiplied by the number of items of the corresponding full-scale.

## 3. Results

**Figure 2:**
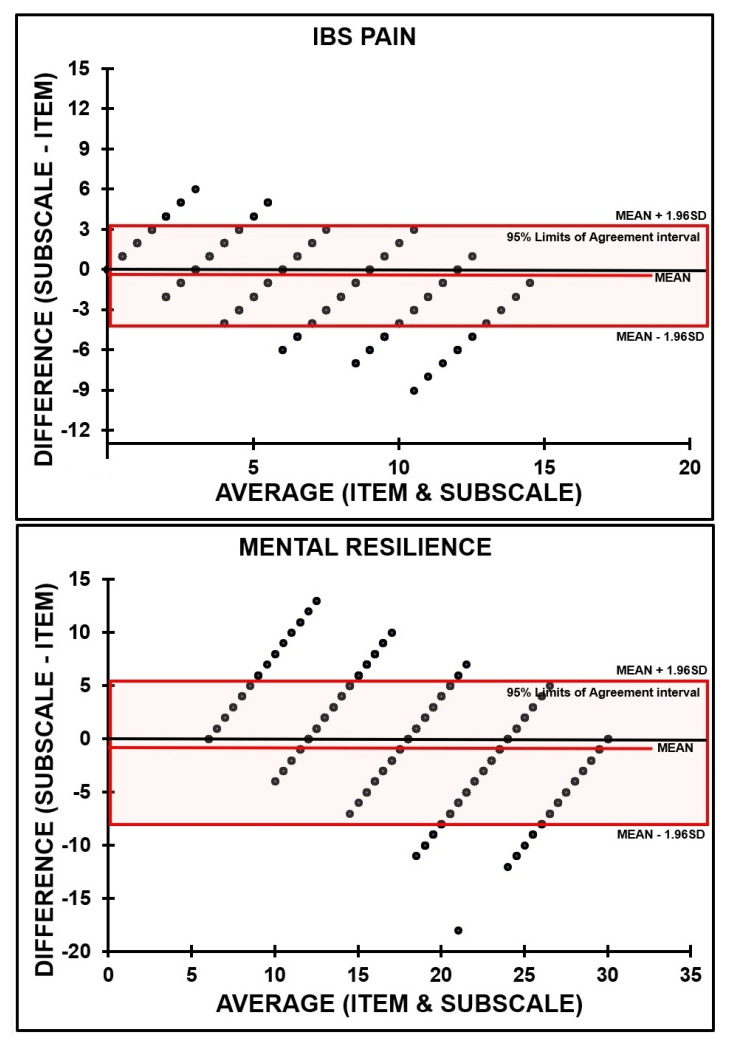

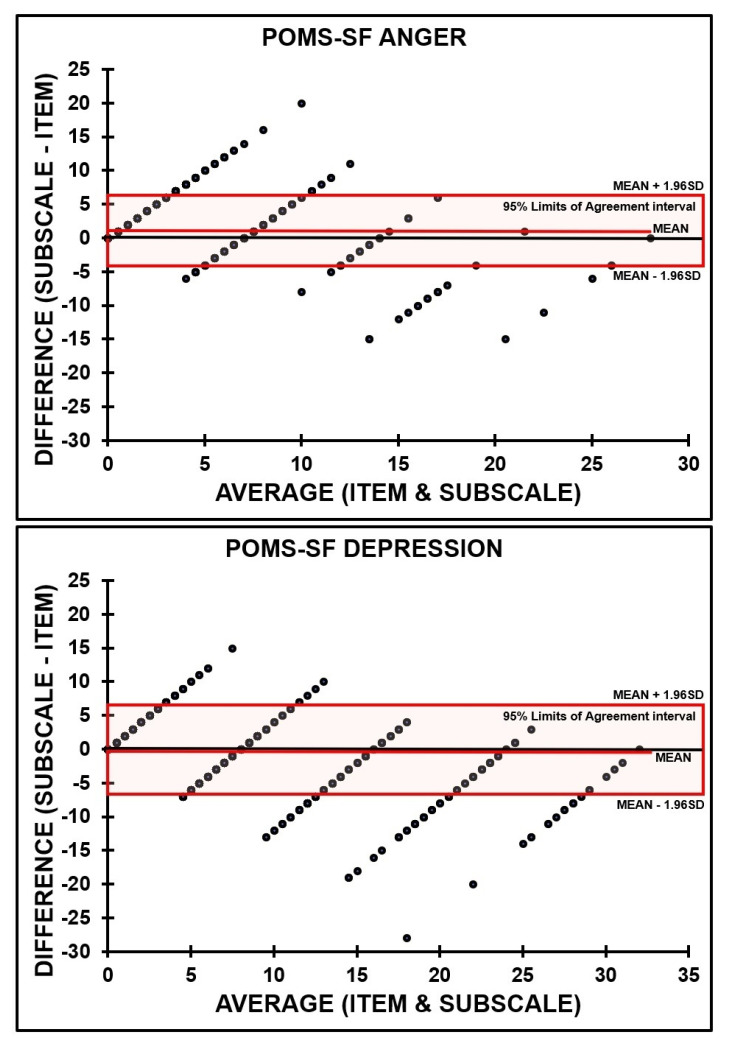

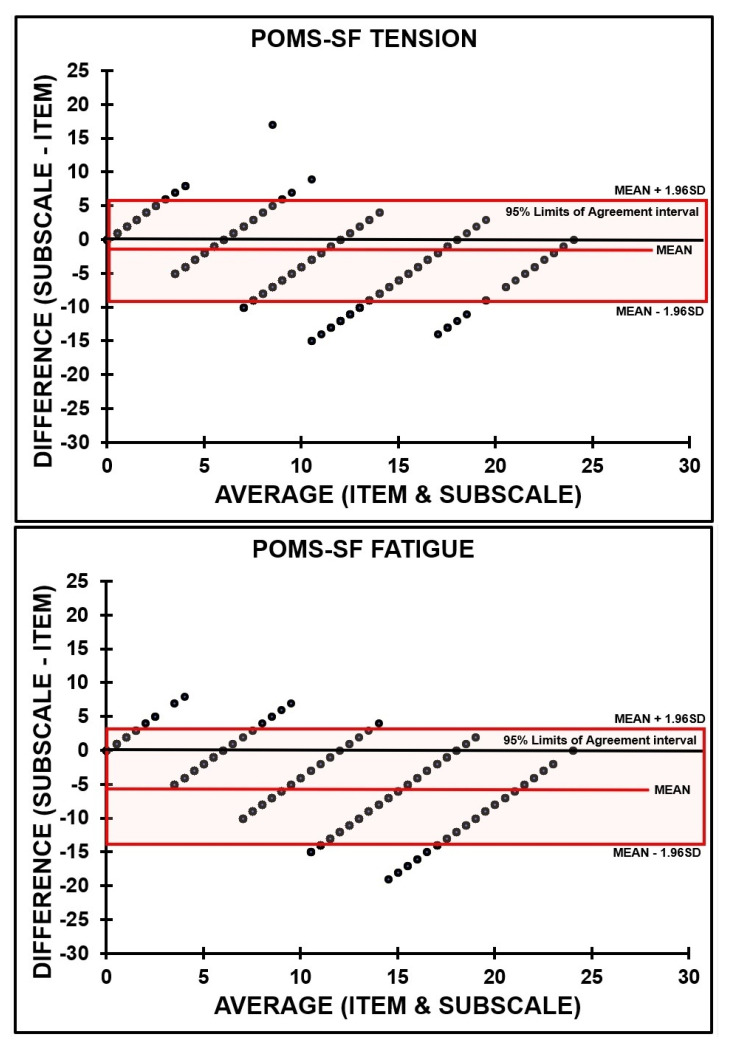

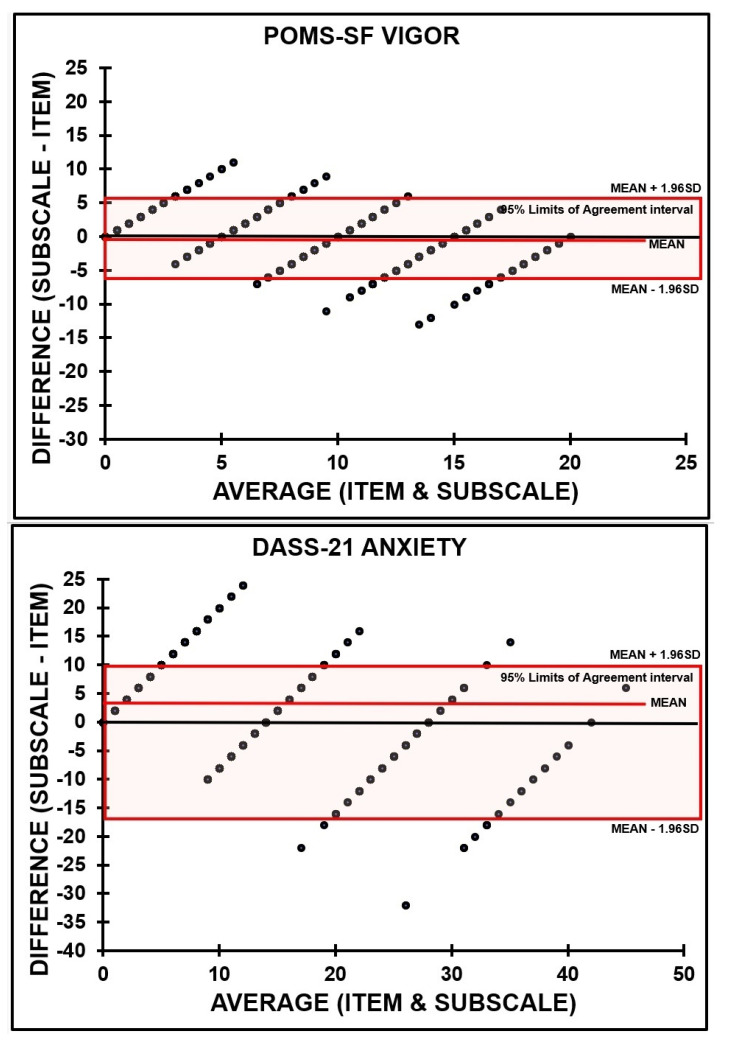

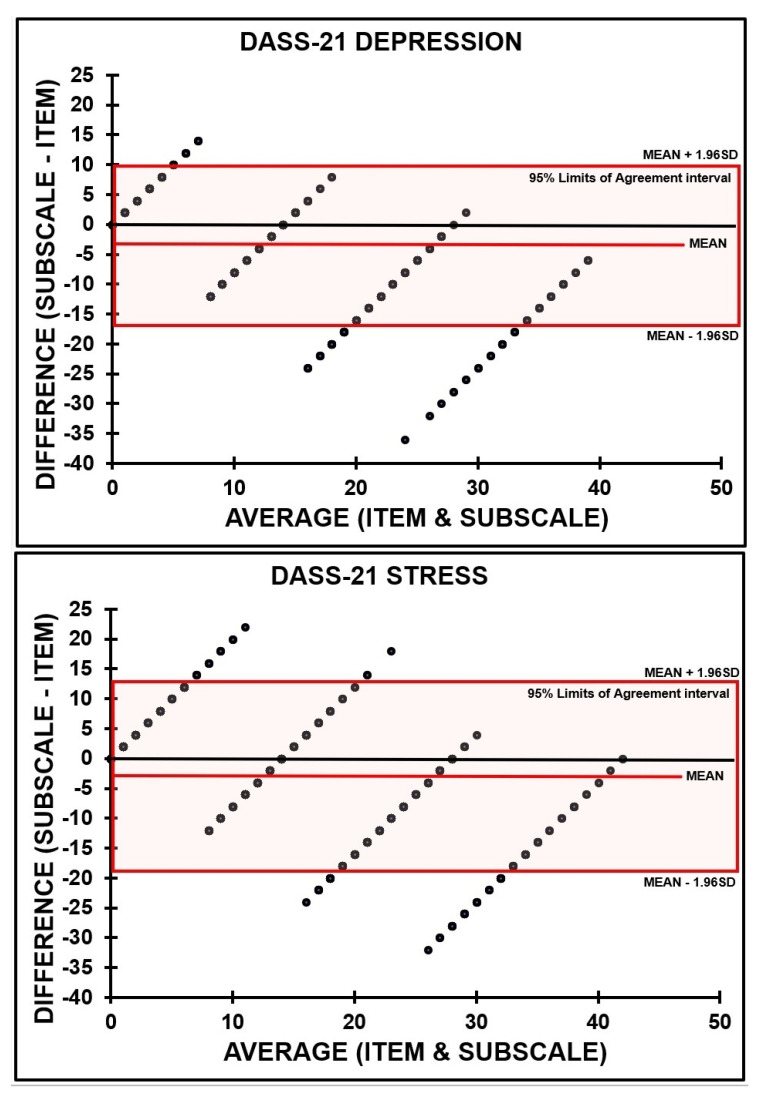
Bland and Altman plots are shown for the irritable bowel syndrome (IBS) subscales constipation, diarrhea, and pain; for mental resilience; for the profiles of mood scales–short form (POMS-SF) subscales anger, depression, tension, fatigue, and vigor; and for the DASS-21 subscales depression, anxiety, and stress. The mean difference (red color line) and 95% limits of agreement (red box) are indicated. Values on the y-axis are the difference between the single-item assessments (multiplied by the number of subscale items) and subscale assessments for each subject against the mean score of the two assessments. Values on the *x*-axis are the average score of the single-item rating and the subscale.

Table 1 summarizes the correlations between the single-item assessments and corresponding subscale and full-scale scores. All correlations were highly significant. Correlations between single-item scores and subscales were considerably higher than correlations between single-item scores and the full-scale scores. Results of the comparisons between single-item scores and subscale scores are summarized in Table 2, and the corresponding Bland and Altman plots in Figure 2. 

It is evident from Table 2 that for most assessments, the single-item was equally effective as the corresponding subscale, as the percentage of difference scores outside the 95% limits of agreement was below 5%. No agreement between single-item and subscale scores was found for POMS-SF anger (Table 2).

Table 3 summarizes the results for the comparisons between single-item scores and full-scale scores. No agreement was found for IBS-constipation and diarrhea; for POMS-SF-anger, depression, and tension; for DASS-21 for anxiety and depression and for sleep initiation and maintenance problems.

## 4. Discussion

To enable mobile, user-friendly assessments, it is desirable that these are short, reliable, and valid. The comparisons between single-item assessments and their corresponding multiple-item subscales in the current study reveal that the vast majority of single-item assessments are in agreement with their lengthier multiple-item subscales. With one exception (POMS-SF anger), it was consistently shown that single-item assessments of unipolar constructs yield similar results to their corresponding more elaborate multiple-item scale.

Agreement between single-item ratings and the full-scales was usually not observed. This is understandable as these scales are composed of several subscales (e.g., POMS-SF and DASS-21) or the scale assesses different constructs that were not represented by a single-item (e.g., sleep initiation and sleep maintenance problems in the SLEEP-50 insomnia subscale). This observation reflects the importance of the fact that a single-item assessment should fully describe all aspects of the construct under investigation. When selecting items from the original scales, the aim was to select the item that best described the construct. For the subscales, this objective was usually achieved, and the single-item univocally described the construct under investigation (e.g., depression and anxiety). However, when assessing constructs that have multiple components (e.g., insomnia or ADHD), assessing only one of these characteristics is usually insufficient to achieve agreement between the single-item rating and multiple-item scale. 

Assessing a construct by composing a multiple-item scale may be problematic in itself. This has been observed in studies that aim to assess a construct by combining ratings of symptoms associated with the construct under investigation. For example, in alcohol hangover research, 47 different hangover symptoms have been identified [18], which have a differential impact on cognitive functioning, physical functioning, and mood [19]. There are currently three validated scales that are commonly used, which assess overall hangover severity by calculating the sum of individual items that rate the frequency and/or severity of specific hangover symptoms such as fatigue, nausea, and headache [20,21,22]. Given the large number of known hangover symptoms, it can be questioned whether the composite scale score adequately represents the overall hangover experience. Indeed, recent research revealed that outcomes of single-item assessments of hangover severity are not in agreement with multiple-item assessments [23].

This observation underlines the fact that single-item ratings may be superior over multiple-item ratings, as these are thought to assess the complete experience of the construct under investigation. That is, all aspects deemed relevant to the patient are included in this assessment, including the presence, severity, and impact of symptoms and experiences, opposed to multiple-item scales which may be at risk of not consulting about certain issues that may be relevant to the subject’s evaluation. Therefore, especially in case of complex constructs, assessments are preferred to be made via direct single-item assessments. Such single-item assessments are ideal for quick time assessments in real-time, which is a clear advantage when using mobile technology, but also in clinical practice when quick results are required, and in clinical trials with time constraints. If more in depth information about a construct is needed, multiple-item assessments or interviews can be conducted at a later stage. Alternatively, a single-item assessment can advise if a more detailed assessment is needed at this point of time.

According to Cohen [24], most correlations between single-item assessments and full-scale outcomes listed in Table 1 can be considerate as moderate to high (r > 0.5). However, the fact that correlations are highly statistically significant is no proof that two assessments are in agreement, i.e., measuring the same construct with an identical outcome. There are many examples of high and significant correlations between measures that actually assess a different construct. Eminent examples are the correlations between bodyweight and height, or between alcohol consumption and smoking [25]. Therefore, one should never rely on correlations to determine if two measures assess the same construct. Instead, the 95% limits of agreement method by Bland and Altman is considered as gold standard to determine if two assessments are in agreement or not [16]. 

A limitation of the current single-item assessments is the fact that these were all conducted with the Likert scales used in the corresponding full subscales, allowing limited differentiation between the answering possibilities. To allow more differentiation in scoring, for future use, it is proposed to apply an 11-point rating scale for the single-item assessments, ranging from 0 (absent) to 10 (extreme severe).

Bland and Altman [16] stated that “the decision about what an acceptable agreement is a clinical one; statistics alone cannot answer the question”. We concur with this viewpoint. It should always be judged whether a single-item rating provides sufficient information in a specific context. For example, it can be argued that complex constructs such as schizophrenia or ADHD can never be accurately represented by a single-item assessment (or single biomarker), and therefore require more thorough and elaborate assessments per se, including assessments with traditional multiple-item questionnaires. Similarly, the single-item human immunodeficiency virus (HIV) risk stage-of-change measure has been shown to disagree with a conventional measure and thus was not reflective of the change of HIV risk [26]. In this study, the conventional measure and the single-item rating did not correspond. It was concluded that the measures do not assess the same construct, and that the single-item assessment needed to be revised or its use should be abandoned. Additionally, a survey from 21 countries compared single-item self-assessment against a multi-item health measure, only the multi-item measure showed a significant correlation with life expectancy [27]. However, the conclusions of this study were based on correlational analysis and the Bland and Alman 95% limits of agreement method was not applied. Nevertheless, clinical judgement based on the purpose of the study should guide the choice of single-item versus multi-item instrument. 

Finally, there is a likelihood that people would respond differently to a single item presented in isolation than to the same item presented in the context of a larger scale composed of conceptually overlapping items. As stated in the introduction, single-item assessments incorporate the subjects’ evaluation of the presence, severity, and impact of a condition, with greater subject-focused information value than the specific symptom-based sum score of multiple-item questionnaires can provide [4]. On the other hand, other literature suggests that multipole-item assessments should be favored as they have a significantly greater accuracy when screening or diagnosing patients [28]. In addition, psychometric theory has suggested that multiple item scales should be preferred as possible measurement error averages out when multiple item scores are summed to obtain a total score [29]. However, this was not evident from the current analysis, as the Bland–Altman comparisons usually showed agreement between single- and multiple item assessments. Taken together, it depends on the purpose of the research study or clinical assessment made whether single-item or multipole item measurements should be preferred.

## 5. Conclusions

In conclusion, the current analysis shows that single-item ratings for irritable bowel syndrome, depression, mood, mental resilience, and insomnia adequately represent the outcomes of traditional multi-item assessments.

## Figures and Tables

**Table 1:** Correlation between single-item assessments multiple-item assessments.

		Correlation with Subscale		Correlation with Full-Scale	
Assessment	N	Correlation	*p*-Value	Correlation	*p*-Value
IBS—constipation	1950	0.728	*p* < 0.0001	0.614	*p* < 0.0001
IBS—diarrhea	1950	0.699	*p* < 0.0001	0.529	*p* < 0.0001
IBS—pain	1950	0.888	*p* < 0.0001	0.694	*p* < 0.0001
POMS-SF Anger	1838	0.477	*p* < 0.0001	0.389	*p* < 0.0001
POMS-SF Depression	1826	0.743	*p* < 0.0001	0.625	*p* < 0.0001
POMS-SF Tension	1828	0.828	*p* < 0.0001	0.762	*p* < 0.0001
POMS-SF Fatigue	1836	0.871	*p* < 0.0001	0.719	*p* < 0.0001
POMS-SF Vigor	1830	0.851	*p* < 0.0001	−0.048	0.042
DASS-21 Anxiety	1706	0.623	*p* < 0.0001	0.586	*p* < 0.0001
DASS-21 Depression	1709	0.800	*p* < 0.0001	0.705	*p* < 0.0001
DASS-21 Stress	1698	0.762	*p* < 0.0001	0.681	*p* < 0.0001
Mental resilience	2075	-	-	0.793	*p* < 0.0001
Sleep initiation problems	2041	-	-	0.739	*p* < 0.0001
Sleep maintenance problems	2041	-	-	0.630	*p* < 0.0001

Spearman’s rank correlations coefficients (Rho) are presented, which are considered significant if *p* < 0.05 (two-tailed). As not all of the subjects completed the full survey, the N is given for each correlation. Abbreviations: DASS-21 = depression anxiety stress scale 21 items, POMS-SF = profiles of mood states short form, IBS = irritable bowel syndrome. - = questionnaire has no subscales.

**Table 2:** Mean (SD) of single-item and subscale assessments.

Assessment	Single-Item Score (x Number of Subscale Items)	Subscale Score (Sum of Subscale Items)	DIFF Score (SD_DIFF_)	95% Limits of Agreement Interval	% of DIFF scores outside the 95% Limits of Agreement interval	Agreement between Methods
IBS—constipation	1.9 (3.2)	3.3 (3.0)	1.5 (1.8)	−2.1, 5.0	1.5 %	Yes
IBS—diarrhea	4.5 (5.0)	4.1 (3.1)	−0.5 (3.3)	−6.9, 6.0	4.9 %	Yes
IBS—pain	4.0 (3.8)	3.4 (2.8)	−0.6 (1.9)	−4.3, 3.3	3.1 %	Yes
POMS-SF Anger	0.9 (3.4)	2.4 (3.5)	1.4 (2.7)	−3.9, 6.7	5.1 %	No
POMS-SF Depression	3.7 (6.9)	3.5 (5.3)	−0.1 (3.5)	−7.0, 6.7	4.8 %	Yes
POMS-SF Tension	6.1 (6.5)	4.2 (4.5)	−1.9 (3.7)	−9.1, 5.4	4.1 %	Yes
POMS-SF Fatigue	10.7 (7.3)	5.3 (5.0)	−5.4 (4.4)	−14.0, 3.2	3.9 %	Yes
POMS-SF Vigor	9.2 (5.6)	9.1 (4.4)	−0.1 (3.0)	−6.0, 5.8	3.3 %	Yes
DASS-21 Anxiety	4.5 (9.1)	7.5 (7.6)	3.1 (6.0)	−9.9, 15.1	4.8 %	Yes
DASS-21 Depression	9.1 (11.1)	5.7 (6.9)	−3.4 (6.7)	−16.5, 9.7	4.4 %	Yes
DASS-21 Stress	12.3 (12.3)	9.8 (7.8)	−2.5 (8.1)	−18.7, 13.7	4.7 %	Yes
Mental resilience ^1^	20.6 (5.7)	19.6 (4.4)	−1.0 (3.4)	−7.7, 5.6	4.9 %	Yes

Sum (SD) scores of the subscales and single-items are presented. ^1^ = a comparison was made with the full-scale score. The SLEEP-50 insomnia scale was omitted from the table as the scale has no subscales for sleep initiation and sleep maintenance problems.

**Table 3:** Mean (SD) of single-item and full-scale assessments.

Assessment	Single-Item Score (x Number of Full Scale Items)	Full-Scale Score (Sum of All Items)	DIFF Score (SD_DIFF_)	95% Limits of Agreement Interval	% of DIFF Scores Outside the 95% Limits of Agreement Interval	Agreement between Methods
IBS–constipation	6.9 (11.6)	10.8 (7.1)	4.0 (8.6)	−12.9, 20.9	6.0 %	No
IBS–diarrhea	9.9 (11.0)	10.8 (7.1)	0.9 (9.1)	−16.9, 18.7	5.9 %	No
IBS–pain	14.8 (14.0)	10.8 (7.1)	−3.9 (10.2)	−23.9, 16.1	4.6 %	Yes
POMS-SF Anger	4.3 (15.4)	30.7 (16.3)	26.4 (15.8)	−4.64, 57.4	5.4 %	No
POMS-SF Depression	14.7 (27.6)	30.7 (16.3)	16.0 (18.8)	−20.8, 52.8	5.6 %	No
POMS-SF Tension	32.7 (34.5)	30.7 (16.3)	−2.1 (24.2)	−49.5, 45.3	5.1 %	No
POMS-SF Fatigue	47.0 (39.1)	30.7 (16.3)	−26.3 (30.6)	−86.3, 33.7	3.0 %	Yes
POMS-SF Vigor	59.1 (36.0)	30.7 (16.3)	−28.3 (40.8)	−108.3, 51.7	2.8 %	Yes
DASS-21 Anxiety	13.4 (27.4)	23.1 (19.7)	9.8 (19.5)	−28.4, 48.0	5.0 %	No
DASS-21 Depression	27.2 (33.3)	23.1 (19.7)	−4.3 (22.7)	−48.8, 40.2	5.3 %	No
DASS-21 Stress	36.9 (37.0)	23.1 (19.7)	−13.9 (27.3)	−67.2, 39.6	4.7 %	Yes
Sleep initiation problems	17.5 (8.3)	16.4 (5.4)	−1.0 (5.5)	−11.8, 9.7	5.6 %	No
Sleep maintenance problems	13.6 (7.3)	16.4 (5.4)	2.9 (5.3)	−7.5, 13.3	5.2 %	No

Sum (SD) scores of the full-scale and single-items are presented.

## Data Availability

The data presented in this study are available on request from the corresponding author.
